# Time to Detection of Yeast Isolates in Pediatric and Adult Patients with Fungemia and its Relevance to Clinical Profile and Outcome

**DOI:** 10.5005/jp-journals-10071-23108

**Published:** 2019-01

**Authors:** Hena Butta, Raman Sardana, Leena Mendiratta, Anupam Sibal, Vidya Gupta, Rajesh Chawla, Ali Ammar Jafri

**Affiliations:** 1-3 Departments of Microbiology, Indraprastha Apollo Hospitals, New Delhi, India; 4 Departments of Pediatrics, Indraprastha Apollo Hospitals, New Delhi, India; 5 Departments of Neonatology, Indraprastha Apollo Hospitals, New Delhi, India; 6 Departments of Critical Care, Respiratory and Sleep Medicine, Indraprastha Apollo Hospitals, New Delhi, India; 7 Department of Biotechnology, Harlal Institute of Management and Technology, Greater Noida, Uttar Pradesh, India

**Keywords:** Continuous monitoring automated blood culture systems, Time to detection, Yeast

## Abstract

**Context:**

Time to detection (TTD) given by continuous monitoring automated blood culture systems (CMABS) have been found to be a predictor of clinical outcome, drug resistance and type of microorganism in cases of bacteremia but the studies evaluating TTD with respect to fungemia are scarce especially from India.

**Aims:**

To evaluate TTD for yeast isolates in fungal bloodstream infections with respect to the type of yeast isolates, risk factors and outcome and to study yeast susceptibility and distribution of yeast isolates with respect to patient population.

**Materials and methods:**

All blood culture specimens were processed in CMABS. The TTD for yeast isolates were recorded. The identification of yeast and susceptibility testing was done by automated methods. A correlation of TTD was done with respect to prior/concurrent yeast isolates, use of antifungal, risk factors and clinical outcome.

**Results:**

Out of 80 yeast isolates, the maximum was *C. parapsilosis* (26.25%) followed by *C. albicans* (16.25%) and *C. tropicalis* (13.75%). A statistically significant difference in the occurrence of yeasts with early TTD (TTD < = 48 hours) and late TTD (TTD > 48 hours) was found. TTD of *C. glabrata* was significantly longer (p = 0.002) while TTD of *C. tropicalis* was significantly shorter (p = 0.013). There was an observable favorable outcome in shorter TTD (< = 48 hours). *C. albicans* and *C. tropicalis* depicted 100% susceptibility for Azoles, Amphotericin B and Echinocandins.

**Conclusion:**

TTD may be used as both diagnostic and prognostic adjunct in fungal bloodstream infections. This study is a step towards this novel approach. We also emphasize on the importance of speciation of yeast isolates and susceptibility testing.

**How to cite this article:**

Butta H, Sardana R, Mendiratta L, Sibal A, Gupta V, Chawla R, Jafri AA. Time to Detection of Yeast Isolates in Pediatric and Adult Patients with Fungemia and its Relevance to Clinical Profile and Outcome. Indian Journal of Critical Care Medicine, January 2019;23(1):27-30.

## INTRODUCTION

The most common cause of systemic fungal infections in human beings is *Candida* and worldwide *Candida* bloodstream infections are showing a rising trend in recent years. The data from a study of *Candida* bloodstream infection in 27 intensive care units (ICU) in India depicted an incidence of 6.51 cases per 1,000 ICU admissions, equating to ~90,000 cases nationally with 35 to 75% mortality that is about 40,000 deaths.^1^ The timely diagnosis of Candidemia is very important to curtail the morbidity and mortality associated with it. CMABS are the greatest advancement in the early diagnosis of Candidemia. Apart from advantages of CMABS like self-contained modular incubation, agitation, self-monitoring of microbial growth with no need of manual manipulation of blood culture bottles the other usefulness is TTD given by the equipment when the blood culture positivity is indicated. TTD is defined as the time between the start of blood culture bottle incubation to the blood culture positive alert signal (as documented by the monitoring system). In bacterial bloodstream infections, TTD has been evaluated for various aspects like a predictor of catheter-related bacterial sepsis, clinical outcome, drug resistance, type of microorganism.^2-6^ Worldwide, there are very few studies which evaluate TTD with respect to Candidemia. So, keeping this in mind, the present study was planned to isolate yeast from the blood cultures of the patients with suspected bloodstream infections, to record TTD of the blood culture positivity in patients with yeast isolates, to compare the TTD of different *Candida* species, to assess the impact of TTD on clinical outcome, to assess the risk factors associated with these yeast isolates and to study the antifungal susceptibility of these yeast isolates.

## MATERIALS AND METHODS

The study was conducted in the Department of Microbiology at a tertiary health care institute in India, between February 2015 to July 2015. All the blood culture specimens were processed in continuous monitoring automated blood culture systems [BACTEC FX or BACTEC 9120 (Becton Dickinson and Company, US) or BacT Alert (Biomerieux, France)] as per the standard protocol. All the blood culture specimens were submitted in the microbiology laboratory in a timely manner following established protocol, and blood culture bottles were immediately incubated in CMABS. When the positive blood culture signal was given by the system, the blood culture bottle was taken out, and subculture was made on Sheep blood agar, MacConkey agar and Sabouraud's Dextrose agar and a smear made, stained by Gram stain. All the yeast isolates grown from the blood culture sequentially were studied. Repeat yeast isolate from the same patient was not included in the study. For each case, TTD was retrieved from the automated continuous monitoring blood culture systems. The yeast isolates were identified by MALDI-TOF Vitek MS (Biomerieux, France) and/or Vitek-2 cards (YST 21343) (Biomerieux, France). The antifungal susceptibility testing was done by using Vitek-2 cards (AST-YS07). The medical record of the patient was collected from the patient case files. The demographic data which was collected include age, gender, the location of the patient (ward versus ICU); the duration of hospitalization prior to the onset of BSI; the presence of predisposing clinical factors, including neutropenia, the use of peritoneal dialysis or hemodialysis; the presence of central venous catheter and parenteral nutrition. Adverse outcomes (organ failure and in-hospital mortality) occurring during hospitalization were recorded.

The statistical analysis of the results was done by statistical package for the social sciences (SPSS) version 20.0 and R version 3.2.0. A p-value of <0.05 was considered to characterize statistical significance.

## RESULTS

During the study period, a total of 105 yeasts were isolated from the blood culture of the patients. Based on the study selection criteria, out of these105 yeast isolates, 80 were included in the study. Out of 80 isolates, 48 (60%) were isolated from male patients and 32 (40%) were isolated from female patients. Out of these 80 patients with yeast isolates, 51 were admitted in critical care area (ICU/HDU), 28 were admitted inwards and 1 was outpatient who was admitted earlier in the hospital.

**Table 1:** Frequency of occurrence of yeast isolates in blood culture

Type of yeast	Number of isolate (%)
Candida parapsilosis	21 (26.25)
Candida albicans	13 (16.25)
Candida tropicalis	11 (13.75)
Candida glabrata	09 (11.25)
Candida haemulonii complex	08 (10)
Candida pelliculosa	06 (7.5)
Other Candida species	05 (6.25)
Candida guillermondii	02 (2.5)
Candida kefyr	01 (1.25)
Candida krusei	01 (1.25)
Cryptococcus neoformans	01 (1.25)
Rhodotorula mucilaginosa	01 (1.25)
Trichosporon species	01 (1.25)
Total no of isolates	80 (100)

The maximum number of yeast isolates in our study were *C. parapsilosis* (26.25%) followed by *C. albicans* (16.25%) and *C. tropicalis* (13.75%). Table 1 shows the frequency of occurrence of yeast isolates in blood culture during the study period. The maximum number of yeasts were isolated from 17 to 60 years of age group (n = 39, 48.75%) followed by >60 years (n = 24, 30%), 0 to 1 month (n = 09, 11.25%), 1 to 16 years (n = 07, 8.75%) and >1 month to 12 months (n = 01, 1.25%).

The range of TTD for all the yeast isolates was 3.7 hours to 148.2 hours with mean TTD of 42.46 hours. The yeasts isolates were divided into two groups: yeasts with early TTD (TTD £ 48 hours) and yeasts with late TTD (TTD >48 hours). The number of yeast isolates with TTD £48 hours were 54 (67.5%) and TTD > 48 hours were 26 (32.5%). The number of various yeasts with respect to early or late TTD are described in Table 2.

TTD of yeasts with respect to various clinical parameters is shown in Table 3. In our study, the total number of patients who were on total parenteral nutrition (TPN) was six. Amongst these six patients, five showed growth of *Candida parapsilosis*.

The antifungal susceptibility of major yeast isolates in our study is given in Table 4.

## DISCUSSION

### Frequency of Distribution of Yeast Isolates

It is important to know the epidemiology of *Candida* bloodstream infections in a particular health care set-up. In our study, amongst *Candida* species, the isolation of non-albicans *Candida* was more frequent in comparison to *C. albicans* (83.1% *vs.* 16.9%). Amongst non-albicans *Candida*, the isolation of *C. parapsilosis* was maximum (32.8%) followed by *C.* *tropicalis* (17.1%), *C. glabrata* (14.0%), *C. haemulonii* complex (12.5%) and *C. pelliculosa* (9.3%). The type of yeast isolates in a health care setting vary according to the geographical area, type of patient population, various risk factors and the type of health care set-up. In a study by, Caggiano et al., the isolation of *C. albicans* and non-albicans *Candida* species in bloodstream infections was found to be 44.2% and 55.8% respectively. Like our study, they also found *Candida parapsilosis* (62.2%) as the most frequent non-albicans *Candida*.^7^ However, Lee et al. in their study found *Candida albicans* (49%) to be the most frequently isolated species followed by *C. parapsilosis* (22%), *C. tropicalis* (14%) and *C. glabrata* (11%).^8^ In India, Chander et al. found isolation of *C.tropicalis* (40.8%) to be maximum followed by *C. albicans* (29.6%) and *C. glabrata* (18.5%).^9^

**Table 2:** Number of yeast isolates with respect to TTD

*Type of yeast*	*TTD>48 hours*	*TTD<48 hours*	*p-value*
*Albicans*	02	11	0.149
*C. glabrata*	07	02	0.002
*C. tropicalis*	0	11	0.013
*C. parapsilosis*	10	11	0.085
*C. pelliculosa*	0	06	0.076
*C. haemulonii complex*	01	07	0.204
*C. guillermondii*	01	01	-
*C. kefyr*	0	01	-
Other *Candida* species	01	04	-
*Trichosporon* spp.	01	0	-
*Cryptococcus neoformans*	01	0	-
*Rhodotorula mucilaginosa*	01	0	-

**Table 3:** TTD of yeasts with respect to clinical parameters

		*TTD≤48 hrs*	*TTD>48 hrs*	*p-value*
Central venous catheter	Yes	70.7%	78.9%	0.440
	No	29.3%	21.0%	0.434
Prior antifungal therapy	Yes	17.5%	36.8%	0.059
	No	82.5%	63.1%	0.057
Outcome	Discharged	72.9%	60%	0.246
	Expired	27.3%	40%	0.254

### The Frequency of Occurrence of Yeast Isolates in Various Age Groups

In our study, we found statistically significant differences (p <0.0005) in the occurrence of yeast isolates in various age groups, with higher occurrences in the age groups "17-60 years" (n = 39) and ">60 years" (n = 24) than others. This finding was in agreement with other studies.^10,11^ Diekema et al. also found a higher occurrence of Candidemia in 19-64 years (38%) and >64 years (46%) of age-group.^9^ Chang et al. in their study found that out of 96 patients with *Candida* bloodstream infections, 53 (55.2%) were adults.^11^

### TTD in Candidemia

In our study, we found statistically significant (p = 0.002) difference in the proportion of patients with early and late TTD (TTD<=48 hours *vs.* >48 hours respectively). Nunes et al. in their study to evaluate the time to positivity (TTP) of blood cultures in patients with *Candida albicans* BSIs found TTD £36 hours in 56.2% cases.^12^

### TTD with respect to *Candida* species

In our study, amongst all the isolated species of Candida, the TTD of *C. glabrata* was found to be significantly longer (p = 0.002) while the TTD of *C. tropicalis* was found to be significantly shorter (p = 0.013). This finding in our study is corroborated by other studies also which have evaluated TTD with respect to *Candida* species. Lai et al. also found the TTD of *C. glabrata* to be significantly longer and that of *C. tropicalis* to be significantly shorter than the TTP of the other species.^13^ Ben-Ami et al. found TTD of *C. glabrata* to be significantly longer than for other *Candida* species (p = 0.001).^14^ In the study by Park SH et al., the mean TTD of *C. glabrata, C. albicans, C. parapsilosis, and C. tropicalis* was found to be 50.8 hours, 37.5 hours, 35.3 hours and 22.4 hours respectively.^15^ Fernandez et al. found the mean TTD of *C. albicans* to be significantly shorter than *C. glabrata* (p <0.0001).^16^

**Table 4:** Antifungal susceptibility of Candida species

	*C. parapsilosis*	*C. albicans*	*C. tropicalis*	*C. glabrata*
Amphotericin B				
0.5-1.0	93.75%	100%	100%	100%
>2	6.25%	0%	0%	0%
Flucytosine				
<4	80%	100%	100%	83.3%
4-16	20%	0%	0%	16.6%
>16	0%	0%	0%	0%
Fluconazole				
<=2 (S)	61.5%	100%	100%	NA
4 (SDD)	23.0%	0%	0%	NA
>=8 (R)	15.3%	0%	0%	NA
<=32 (SDD)	NA	NA	NA	100%
>=64 (R)	NA	NA	NA	0%
Voriconazole				
<=0.12 (S)	85.7%	100%	100%	85.7%
0.25-0.5 (SDD)	0%	0%	0%	0%
>=1 (R)	14.2%	0%	0%	14.2%
Caspofungin				
<=0.12 (S)	NA	NA	NA	100%
0.25 (I)	NA	NA	NA	0%
>=0.5 (R)	NA	NA	NA	0%
<=0.25 (S)	NA	100%	100%	NA
0.5 (I)	NA	0%	0%	NA
>=1 (R)	NA	0%	0%	NA
<=2 (S)	100%	NA	NA	NA
4 (I)	0%	NA	NA	NA
>=8 (R)	0%	NA	NA	NA
S = Sensitive, R = Resistant, SDD = Susceptible dose dependent, I = Intermediate, NA = Not applicable				

### TTD with Respect to Clinical Parameters

In our study, we did not find a statistical correlation between TTD and central venous catheter-related Candidemia and use of antifungal agent before or concurrent to a collection of specimen. However, Ronen et al. found a statistical correlation between TTD and catheter-related Candidemia.^14^ They found TTD was shorter for definite catheter-related candidemia than that for candidemia from other sources (p < 0.001). The reason for the non-significant correlation between TTD and use of antifungal agent may be a collection of specimen for blood culture just before next due dose of the antifungal drug.

It has been observed that a shorter time to blood culture positivity in automated systems has been associated with worse prognosis in infections caused by bacteria. But the studies analyzing the time of positive *Candida* spp culture are rare. In our study, though there was an observable favorable outcome in shorter TTD (<48 hours), but this observation was not statistically significant. But, Nunes et al. found an association of longer TTD with higher mortality.^12^ Like our study, Ben-Ami et al. did not find a significant correlation between the TTD and in-hospital mortality (p = 0.4). The adverse clinical outcomes like death, shock, respiratory failure, and acute renal failure occurred in similar frequency in patients with long and short TTD.^14^

### Antifungal Susceptibility

In our study, *C. albicans* and *C. tropicalis* showed 100% in vitro susceptibility for Azoles, Amphotericin B and Echinocandins. *C. parapsilosis* showed no resistance for Echinocandins, 6.25% resistance for Amphotericin B and ~15% resistance for azoles. *C. glabrata* showed no resistance for Amphotericin B and Echinocandins and 14.2% resistance for Voriconazole. For Fluconazole all (100%) *C. glabrata* isolates showed M.I.C. in SDD range. In a multicentric study across India by Chakrabarti et al., the resistance rates for *C.parapsilosis* were found to be 4%, 3 to 4% and 0% for Amphotericin B, Azoles and Caspofungin respectively. 98.5% of *C.glabrata* isolates showed M.I.C. in SDD range for Fluconazole. For Amphotericin B and Caspofungin, the resistance rate in*C. glabrata* were 3.1% and 23.1% respectively. *C.tropicalis* showed 1%, 2.6%, 8.1% and 4.2% resistance for Amphotericin B, Fluconazole, Voriconazole, and Caspofungin. *C. albicans* showed 0.5%, 5.2%, 7.8% and 3.6% resistance for Amphotericin B, Fluconazole, Voriconazole, and Caspofungin.^1^

## CONCLUSION

Early recognition of *Candida* bloodstream infection has been associated with improved outcome. It is important to know the changing epidemiology of bloodstream infections. Non-albicans *Candida* is found to be emerging as an important cause of bloodstream infections in all age groups. Amongst non-albicans *Candida*, the maximum isolation of*C.parapsilosis* has been seen in our study. *C.albicans* and *C.tropicalis* have depicted 100% in vitro susceptibility to various antifungal agents in our study. TTD has been found to differ amongst different species of *Candida*, so can be utilized as a predictor of a group of species. Longer TTD values for *Candida* species causing fungemia may be predictive of certain species like *C. glabrata*. Also, Shorter TTD has been observed to be associated with the more favorable outcome though not statistically significant. Hence, TTD can be advocated as a useful diagnostic and prognostic adjunct in cases of candidaemia.

## References

[1] Chakrabarti A,, Sood P,, Rudramurthy SM,, Chen S,, Kaur H,, Capoor M, (2015;). Incidence, characteristics and outcome of ICU-acquired candidemia in India.. Intensive Care Med.

[2] Rogers MS,, Oppenheim BA. (1998;). The use of continuous monitoring blood culture systems in the diagnosis of catheter related sepsis.. J Clin Pathol.

[3] Marra AR,, Edmond MB,, Forbes BA,, Wenzel RP,, Bearman GML. (2006;). Time to Blood Culture Positivity as a Predictor of Clinical Outcome of Staphylococcus aureus Bloodstream Infection.. J Clin Microbiol.

[4] Lai CC,, Wang CY,, Liu WL,, Cheng A,, Lee YC,, Huang YT (2011;). Time to blood culture positivity as a predictor of drug resistance in Acinetobacter baumannii complex bacteremia.. J Infect.

[5] Lai CC,, Wang CY,, Liu WL,, Hou CC,, Huang YT,, Hsueh PR. (2011;). Time to blood culture positivity as a predictor of methicillin resistance in Staphylococcus aureus bacteremia.. J Infect.

[6] Ruimy R,, Armand-Lefevre L,, Andremont A. (2005;). Short time to positivity in blood culture with clustered gram-positive cocci on direct smear examination is highly predictive of Staphylococcus aureus.. Am J Infect Control.

[7] Caggiano G,, Coretti C,, Bartolomeo N,, Lovero G,, De Giglio O,, Montagna MT. (2015;). Candida Bloodstream Infections in Italy: Changing Epidemiology during 16 years of Surveillance.. BioMed Research International.

[8] Lee JS,, Shin JH,, Lee K,, Kim MN,, Shin BM,, Uh Y, (2007;). Species distribution and susceptibility to azole antifungals of candida blood stream isolates from eight university hospital in Korea.. Yonsei Med.

[9] Chander J,, Singla N,, Sidhu SK,, Gombar S. (2013;). Epidemiology of Candida blood stream infections: experience of a tertiary care centre in North India.. J Infect Dev Ctries.

[10] Diekema DJ,, Messer SA,, Brueggemann AB,, Coffman SL,, Doern GV,, Herwaldt LA, (2002;). Epidemiology of Candidemia: 3-Year Results from the Emerging Infections and the Epidemiology of Iowa Organisms Study.. J Clin Microbiol.

[11] Chang MR,, Correia FP,, Costa LC,, Xavier PCN,, Palhares DB,, Taira DL, (2008;). Candida bloodstream infection: data from a teaching hospital in matogrosso do sul, Brazil.. Rev Inst Med trop S Paulo.

[12] Nunes CZ,, Marra AR,, Edmond MB,, Victor EDS,, Pereira CAP. (2013,). Time to blood culture positivity as a predictor of clinical outcome in patients with Candida albicans bloodstream infection.. BMC Infectious Diseases.

[13] Lai CC,, Wang CY,, Liu WL,, Huang YT,, Hsueh PR. (2012;). Time to positivity of blood cultures of different Candida species causing fungaemia.. J Med Microbiol.

[14] Ben-Ami R,, Weinberger M,, Orni-Wasserlauff R,, Schwartz D,, Itzhaki A,, Lazarovitch T, (2008;). Time to blood culture positivity as a marker for catheter related candidemia.. J Clin Microbiol.

[15] Park SH,, Shim H,, Yoon NS,, Kim MN. (2010;). Clinical relevance of time-to-positivity in BACTEC9240 blood culture system.. Korean J Lab Med.

[16] Fernandez J,, Erstad BL,, Petty W,, Nix DE. (2009,). Time to positive culture and identification for Candida blood stream infections.. Diagn Microbiol Infect Dis.

